# Association of sleep hygiene knowledge and physical activity with sleep quality in nursing and medical students: a cross-sectional study

**DOI:** 10.3389/fspor.2025.1453404

**Published:** 2025-03-20

**Authors:** Jacksaint Saintila, David Javier-Aliaga, Norma del Carmen Gálvez-Díaz, Luz Antonia Barreto-Espinoza, Noemi A. Buenaño-Cervera, Yaquelin E. Calizaya-Milla

**Affiliations:** ^1^ Research Group for Nutrition and Healthy Behaviors, Universidad Señor de Sipán, Chiclayo, Perú; ^2^ Escuela de Psicología, Universidad Continental, Lima, Perú; ^3^ Research Group for Nutrition and Lifestyle, Universidad Peruana Unión, Lima, Perú

**Keywords:** IPAQ, medical students, nursing students, physical activity, PSQI, sleep quality, university students

## Abstract

**Background:**

Sleep quality and physical activity are fundamental factors in the optimal functioning of the human organism and maintaining overall health. This study aimed to evaluate the relationship between knowledge of sleep hygiene and physical activity in relation to sleep quality among nursing and medical students.

**Methods:**

In a cross-sectional online study, a suitable and convenient sample of 300 nursing and medical students from a private university located in Chiclayo, Peru, was collected. Data were collected using the following instruments: a questionnaire concerning sleep hygiene knowledge, the International Physical Activity Questionnaire (IPAQ), and the Pittsburgh Sleep Quality Index (PSQI). The data collection period spanned January and February 2024.

**Results:**

Of the total sample, 59.3%, 51.7%, and 69.7% reported insufficient knowledge of sleep hygiene, low physical activity and poor sleep quality, respectively. Sleep hygiene knowledge and physical activity explained 17.9% of the variability in sleep quality (*R*^2^ = 0.179, *F* = 32.31, *p* < 0.001). In addition, there is a positive and significant association between knowledge of sleep hygiene (*β* = 0.27; *p* < 0.001) and physical activity (*β* = 0.24; *p* < .001) with sleep quality.

**Conclusion:**

The findings indicate that interventions designed to improve sleep hygiene knowledge and physical activity may be effective strategies for improving sleep duration among nursing and medical students.

## Introduction

Adequate sleep is a biological condition that plays an essential role in the optimal functioning of the human organism and the maintenance of health (1). In fact, during sleep, several fundamental processes occur in the body that can have positive impacts on cognitive function, academic performance, and the physical and mental health of university students (2). Good sleep quality includes aspects that reflect both the quantity and quality of rest; and are commonly used in research and clinical evaluations to determine the quality of sleep of a person (3). These include a sleep latency of less than 30 min, the number of awakenings exceeding five minutes, a shorter wake-up time after sleep onset, sleep efficiency or sleep duration (between seven and nine hours per night), subjective sleep quality, and sleep regularity (3, 4). However, poor sleep quality represents a significant global public health concern (1). The prevalence of poor sleep quality is 47.12%, while insomnia symptoms are present in 21.15% of the population (5).

Sleep quality is frequently impaired in health science professionals and young adults, including university students; this is particularly evident in those pursuing demanding careers such as nursing and medicine (6, 7). Indeed, the university experience of these students is marked by a multitude of challenges that extend beyond the conventional boundaries of academic pursuits (8, 9). For example, students in these fields face a combination of intense academic workloads, long hours of practical training, emotional exposure to clinical environments, and the need for high levels of responsibility and decision-making under pressure (10, 11). Additionally, the requirement for extended clinical rotations, which often overlap with academic obligations, adds to the overall stress and time demands unique to health science programs (12). Additionally, the university represents a transition stage in which students transition from structured preparatory courses and high school attendance to a different phase, marked by the diverse academic activities of higher education (8). This phase is characterized by intensive daily study routines and a considerable increase in the academic load, which can impact conventional sleep schedules (13).

Sleep disorders, such as insomnia, excessive daytime sleepiness, and circadian rhythm disruptions, are highly prevalent among university students and significantly impact their health-related quality of life (9). Studies estimate that approximately 50%–60% of university students experience poor sleep quality, which is often associated with heightened stress levels, academic pressures, and irregular sleep schedules (14). These sleep disturbances impair cognitive functioning, memory, and academic performance and increase the risk of developing long-term health conditions such as depression, anxiety, and cardiovascular diseases (15). Moreover, poor sleep quality strongly correlates with reduced health-related quality of life, limiting students' ability to perform daily activities and maintain emotional well-being (16). Addressing these issues is critical for improving the academic and personal outcomes of university students.

Therefore, it is important to understand the factors that affect sleep quality in this population to develop interventions that can improve their well-being and academic performance.

On the other hand, lack of knowledge of sleep hygiene represents a significant barrier to achieving adequate rest and represents an essential aspect of research on sleep quality (17). Sleep hygiene knowledge, which encompasses practices such as maintaining regular bedtime and wake-up times, avoiding caffeine before bedtime, and creating a sleep-friendly environment, has also been associated with improved sleep quality. For example, some studies have shown that increased awareness of these recommendations can lead to significant improvements in sleep quality (17–19). However, the results of the investigation have not been conclusive and there is no clear correlation between individual knowledge of sleep hygiene and improved sleep quality (15, 20). Therefore, addressing the knowledge gap is important to improve the sleep quality of university students, especially those in demanding fields of study such as health sciences.

In relation to physical activity, it is important to note that it has several health benefits, including improved sleep quality (21). In fact, lack of physical activity represents a significant threat to health, with the potential to induce stress, depression, anxiety, and suboptimal academic performance (22, 23). Regular physical activity has been associated with improvements in various aspects of sleep in university students, including sleep latency, total sleep duration, sleep efficiency, and reduced nighttime awakenings (24). In the university setting, particularly those in demanding programs such as nursing and medicine, adequate physical activity can serve as an effective strategy to improve sleep quality. For example, a study conducted in this specific population demonstrated that lack of moderate physical activity can negatively affect sleep (25).

Mechanisms underlying the positive association between physical activity and sleep quality can include the regulation of circadian rhythm, the reduction of stress and anxiety, and the promotion of healthy fatigue, which facilitates the onset of sleep (26). Physical activity has been shown to enhance the release of serotonin, a neurotransmitter that contributes to the regulation of mood and the sleep-wake cycle, and melatonin, a hormone that promotes sleep onset and maintenance. These effects collectively contribute to improving the quality and regularity of sleep (21, 26). However, while physical activity in general is beneficial for sleep quality, excessive physical activity can have the opposite effect and act as an additional stressor, reducing sleep efficiency by dysregulating the hypothalamic-adrenal axis (27). Consequently, encouraging sufficient physical activity may be an effective approach to improving sleep, particularly in populations experiencing elevated levels of stress and academic demands, such as nursing and medical students.

Studying the factors that affect sleep quality in nursing and medical students is particularly important given the unique demands and challenges they face. These students must not only meet academic requirements but also be exposed to high-pressure situations in clinical settings. A lack of sufficient sleep can affect an individual's ability to make prompt and precise decisions, which is important in the field of healthcare. Insufficient or poor quality sleep has been associated with a reduction in concentration, memory, and cognitive abilities, which, in turn, increases the risk of medical errors that can have serious consequences for patients. Therefore, this study aimed to determine the association between physical activity and sleep hygiene knowledge with sleep quality in nursing and medical students.

## Materials and methods

### Design

A cross-sectional study was conducted (28). We explored the influence of physical activity and sleep-related knowledge on sleep quality in nursing and medical students at a private university in Peru.

### Participants of the study

Data were collected between January and February 2024 through an online survey questionnaire at a private university located in Chiclayo, Peru. The survey was developed and administered through SurveyMonkey on-line survey software. Participants were contacted through institutional email. Participants were selected by non-probability convenience sampling (28, 29). The study included students who were enrolled at the time of the study, regular students (those with more than 12 credits), who provided informed consent, and those who completed the entire survey. Those who did not adequately answer the questions in the questionnaire were excluded. Therefore, a total of 300 university students were considered in this study.

The sample size was calculated using Soper's Free Statistics Calculators (30). The number of explanatory variables in the multiple linear regression is two. For an effect size of 0.10, a statistical power of 0.90, and a significance level (*α*) of 0.05 for multiple regression analysis, the minimum sample size required was 129.

### Ethical aspects

Before the start of the online data collection, electronic informed consent was obtained from all participants. The research protocol was approved by the Research Ethics Committee of the Faculty of Health Sciences of the Universidad Señor de Sipán. The study was carried out in accordance with the ethical principles set forth in the Declaration of Helsinki and its subsequent amendments.

### Variable measurement

#### Physical activity

The level of physical activity was assessed using the short version of the International Physical Activity Questionnaire (31). The instrument comprises a total of seven questions, six of which enquire about the number of days and duration of time (in minutes) spent in vigorous and moderate intensity physical activity and walking in the previous seven days (32). The seventh question of the questionnaire enquires about the number of hours the participant was seated during the previous week. The test-retest reliability of the instrument is adequate, with a correlation coefficient of up to 0.79 (33). Additionally, in the context of Latin America (34), the instrument demonstrated an intraclass correlation coefficient ranging from 0.56 to 0.89, indicating a level of agreement from moderate to almost perfect (35).

The IPAQ is considered one of the best instruments for assessing physical activity in university students (36). Similarly, physical activity was classified into three categories according to the methodology of the International Physical Activity Questionnaire:

- High: High intensity activity on at least three days that accumulates a minimum of 1,500 metabolic equivalents-minutes/week. Seven or more days of any combination of physical activity that accumulates at least 3,000 metabolic equivalents minutes/week.
- Moderate: Three or more days of high intensity activity of at least 20 min per day. Five or more days of moderate intensity activity and/or walking for at least 30 min daily. Five or more days of any combination of walking, moderate, and high intensity activity that achieves a minimum of 600 metabolic equivalents-minutes per week.
- Low: The low activity level includes cases not included in categories 1 and 2.

#### Knowledge of sleep hygiene

Sleep hygiene knowledge was measured using a questionnaire developed in a previous study based on available literature (17). Sleep hygiene knowledge refers to understanding the fundamental aspects that influence sleep quality. This includes maintaining regular bedtimes, controlling the length of daytime naptimes, avoiding distractions before bedtime, and properly preparing the bedroom, ensuring adequate temperature and humidity (17). The questionnaire consisted of 10 multiple-choice questions, in which participants had to select a single correct answer from five available options. For example, “What is the optimal humidity in the room where you sleep?”, with the following response options: (a) approximately 30% (b) about 40% (c) approximately 50% and (d) I don't know. Each correct answer scored 1 point, while incorrect answers scored no points. The scores obtained ranged from 0 to 10 points and the knowledge of the respondents was classified into three levels: insufficient (0–3 points; 0%–32%), sufficient (4–6 points; 36%–64%), and good (7–10 points; 68%–100%). We evaluated the instrument in our setting considering a total of 36 university students in nursing and medicine, who were subsequently excluded from the study. With respect to reliability, a Cronbach alpha of 0.78 was obtained.

#### Sleep quality

To assess sleep quality, the Spanish version of the Pittsburgh Sleep Quality Questionnaire (PSQI) was used (37). This instrument, which has been specifically designed to measure sleep quality, comprises 19 questions that have been divided into seven components. Each component is scored on a scale of 0–3. The seven components of the questionnaire are presented in the following order: The first component of the questionnaire concerns subjective sleep quality, second sleep latency, third sleep duration, fourth sleep efficiency, fifth sleep disturbances, sixth use of sleep medications and seventh daytime dysfunction (37). These components are summed to obtain a total score ranging from 0 to 21, classifying the result into two categories: scores <5 indicate “good sleep quality” and >5 indicate “poor sleep quality” (37). In addition, the reliability of the instrument has been validated, showing a high internal consistency with a Cronbach's α of 0.81 (38). In the Peruvian population, the instrument demonstrated internal consistency with a Cronbach's alpha of 0.564 (39).

#### Sociodemographic data

Participants reported demographic data such as age, sex, residence, monthly income, religion, and career.

### Statistical analysis

Sociodemographic variables were analyzed using frequency tables and percentages. For the association analysis, a multiple linear regression method was applied to evaluate the relationship between the independent variables (sleep hygiene and physical activity) and sleep quality (dependent variable). A significance level of 5% was established to ensure the reliability and validity of the results. The data were processed and analyzed using the statistical software SPSS version 25 (SPSS Inc., Chicago, IL, USA).

## Results

Table 1 presents the descriptive analysis of the sociodemographic characteristics, physical activity levels, sleep hygiene knowledge, and sleep quality among 300 nursing and medical students. The mean age of participants was 25.06 ± 4.02 years, with the majority being female (67.7%) and residing in urban areas (94%). Regarding household income, 52.3% reported a monthly income between $580.81 and $2,904.32 USD. The sample was composed of 38.4% nursing students and 61.6% medical students. In terms of sleep hygiene knowledge, 59.3% of participants demonstrated insufficient knowledge, while 36.7% reported sufficient levels, and only 4% achieved a good level. Physical activity levels were predominantly low (51.7%), with 46% reporting moderate activity and just 2.3% engaging in high physical activity. Sleep quality, assessed via the PSQI, revealed that 69.7% of students experienced poor sleep quality (PSQI >5), whereas 30.3% reported good sleep quality (PSQI ≤5).

**Table 1:** Descriptive analysis of sociodemographic characteristics, physical activity, knowledge of sleep hygiene, and sleep quality.

Characteristics	*n*	%
Age (M ± SD)	25.06 ± 4.02	
Sex		
Female	203	67.7
Male	97	32.3
Place of residence		
Urban	282	94.0
Rural	18	6.0
Monthly family income (USD)^a^		
<580.81	87	29.0
580.81–2,904.32	158	52.7
>2,904.32	55	18.3
Academic discipline		
Nursing	115	38.4
Medicine	185	61.6
Hygiene knowledge		
Insufficient	178	59.3
Sufficient	110	36.7
Good	12	4.0
Hygiene knowledge (M ± SD)	6.06 ± 1.38	
Physical activity		
Low	155	51.7
Moderate	138	46.0
High	7	2.3
Sleep quality		
PSQI >5 (poor quality)	209	69.7
PSQI ≤5 (good quality)	91	30.3
Sleep quality (M ± SD)	7.05 ± 2.02	

^a^ The Peruvian Sol (PEN) is the national currency of Peru (ISO code: PEN), as per the ISO-4217 standard. For international reference, the reported monthly household income has been converted to U.S. dollars (USD) using an approximate exchange rate of 1 USD = 3.70 PEN, corresponding to the period of data collection.

Table 2 indicates that physical activity is significantly negatively correlated with sleep quality (*r* = −0.331, *p* < 0.01), suggesting that higher levels of physical activity are associated with better sleep quality (as measured by a lower PSQI score). Additionally, a significant positive correlation was found between hygiene knowledge and sleep quality (*r* = 0.224, *p* < 0.01), indicating that greater knowledge of sleep hygiene is associated with better sleep quality. Furthermore, hygiene knowledge and physical activity also showed a weak but significant positive correlation (*r* = 0.195, *p* < 0.01), suggesting that individuals with higher levels of sleep hygiene knowledge tend to engage in more physical activity.

**Table 2:** Correlation analysis of study variables.

Variables	Physical activity	Hygiene knowledge	Sleep quality
Physical activity	1		
Hygiene knowledge	0.195^a^	1	
Sleep quality	−0.331^a^	0.224^a^	1

The correlation is significant at the 0.01 level (2-tailed).

^a^ The relationship analysis was performed using Pearson's correlation coefficient.

The multiple linear regression analysis showed that the overall model is significant (F = 32.319, *p* < 0.001) and explains 17.9% of the variability in sleep quality (R^2^ = 0.179, adjusted R^2^ = 0.173). The independent variables included sleep hygiene knowledge and physical activity (Table 3).

**Table 3:** Multiple regression coefficients B (unstandardized), *β* (standardized) and *t*-test.

Model	R	R^2^	Adj R^2^	SE	F	*p*
1	0.423	0.179	0.173	0.434	32.319	<0.001

Independent variables: (constant), knowledge of sleep hygiene and physical activity. SE, standard error of the estimate; R^2^, R-squared; Adj R^2^, adjusted R-squared.

The analysis of regression coefficients indicated that both independent variables were significantly associated with sleep quality (*p* < 0.001). However, sleep hygiene knowledge had a greater effect compared to physical activity, with a standardized beta coefficient of *β* = 0.278 (B = 0.04, *t* = 5.01, *p* < 0.001), while physical activity presented a coefficient of *β* = 0.242 (B = 0.21, *t* = 4.36, *p* < 0.001) (Table 4). This suggests that sleep hygiene knowledge has a slightly stronger impact on sleep quality than physical activity.

**Table 4:** Multiple regression coefficients.

Model		Unstandardized coefficients		Standardized coefficients	*t*	*p*
		B	SE	β		
1	(Constante)	0.58	0.15		3.85	<0.001
	Hygiene knowledge	0.04	0.01	0.28	5.01	<0.001
	Physical activity	0.21	0.05	0.24	4.36	<0.001

Dependent variable: quality of sleep. B = unstandardized regression coefficient; SE = standard error of B; β = standardized regression coefficient; *t* = *t*-test value for the independent variable; *p* = *p*-value for statistical significance. Independent variables: Hygiene knowledge and physical activity.

## Discussion

Sleep quality is critical to the well-being and academic performance of university students, especially those pursuing demanding careers such as nursing and medicine. These students face intense schedules, demanding clinical practices, and a high academic stress load, which can lead to significant sleep problems. Lack of adequate sleep can affect physical and mental health and can compromise your ability to provide quality patient care. This study aimed to examine how knowledge of sleep hygiene and physical activity influence the quality of sleep of these students. The study revealed that both sleep hygiene knowledge and physical activity are significantly associated with sleep quality in nursing and medical students.

The general assessment of sleep quality revealed that more than 69% of nursing and medical students reported poor sleep quality. Furthermore, the mean score was 7.05, indicating poor sleep quality in this population. This may be attributed, in part, to the high prevalence of insufficient knowledge of sleep hygiene and physical inactivity observed in the study population. This finding is consistent with that of a study of nursing and medical students from two countries, which found that 74% of participants reported poor sleep quality (9). Similarly, a recent study conducted in Poland revealed PSQI scores of 6.86 and 7.38 among medical students, indicating suboptimal sleep quality (8). In addition, several studies conducted during the pandemic in nursing and medical students have shown an increase in sleep problems and symptoms of insomnia (40, 41). Therefore, it is recommended that more attention is paid to the high prevalence of poor sleep quality among university students, with a particular focus on those studying health sciences.

The results of the analysis indicated a positive correlation between knowledge of sleep hygiene and sleep quality. This suggests that as the levels of knowledge of sleep hygiene increased, the overall quality of sleep improved. This finding indicates that a more comprehensive understanding of practices that improve sleep quality may have a direct and beneficial impact on sleep patterns in nursing and medical students. This result is consistent with previous studies that have demonstrated the effectiveness of sleep hygiene education in improving sleep quality. For example, a study of young adults revealed a positive and statistically significant correlation between knowledge about sleep hygiene and sleep quality (17). Similarly, a systematic review of studies conducted in university students highlighted that educational programs focused on sleep hygiene can be an effective intervention to improve sleep quality (42). However, our findings diverge from those of a study involving 87 medical students. This discrepancy may be attributed to the smaller number of participants in the aforementioned study (43).

It is important to emphasize that lack of knowledge about sleep hygiene can have considerable negative effects. First, students who are unfamiliar with the principles of sleep hygiene may adopt habits that impede their ability to fall and stay asleep. For example, the use of electronic devices in the hours preceding bedtime can suppress the production of melatonin, the hormone that regulates sleep. This can result in prolonged sleep latency and a reduction in the quality of rest (44). Furthermore, a lack of awareness about the importance of an optimal sleeping environment, including the need for a dark, quiet, and cool room, can result in conditions that are incompatible with rest (45). This can result in increased nighttime awakenings and reduced sleep efficiency, which in turn can contribute to daytime fatigue and decreased academic and professional performance (9, 46, 47). Given these consequences, it is necessary for educational programs to incorporate sleep hygiene components to equip students with the necessary tools to better manage their rest. Improving sleep quality confers numerous benefits on students, including improved physical and mental health, improved learning and memory, reduced clinical errors, and greater resilience to academic stress (48, 49).

Another pertinent finding is that physical activity is positively and significantly associated with the quality of student sleep. This finding indicates that students who engage in regular physical activity tend to experience improved sleep quality. This result is consistent with numerous previous studies that have documented a positive relationship between physical activity and sleep quality (50–52). For example, a study of 2,626 university students in Brazil revealed that those who engaged in sufficient physical activity during their leisure time exhibited lower scores on the global PSQI, which is a measure of sleep quality (50). A similar study conducted with university students in Saudi Arabia revealed a significant association between PSQI global scores and physical activity levels (51). Moreover, these findings were corroborated by another study that examined young adults, which also demonstrated a positive correlation between physical activity and sleep quality (52). These studies reinforce the notion that physical activity not only improves overall health, but also plays a fundamental role in optimizing sleep quality. This underscores the importance of integrating regular exercise habits into the daily routines of college students and young adults.

There are several mechanisms by which physical activity can improve sleep quality. For example, it facilitates the regulation of circadian rhythm, which is the internal biological clock that determines sleep-wake cycles by increasing melatonin production (26). By better aligning the circadian rhythm with the natural light-dark cycle, physical activity facilitates the onset of sleep and improves the continuity of sleep. Furthermore, physical activity has been shown to reduce stress and anxiety levels, which are frequently associated with sleep disturbances (21). Exercise releases endorphins and other neurotransmitters that promote a sense of well-being and relaxation, which can help you fall asleep and stay asleep throughout the night (21). Another mechanism is the promotion of healthy fatigue. Physical activity increases energy expenditure during the day, which can result in increased fatigue at the end of the day. This, in turn, facilitates the transition to deep, restful sleep (53). Furthermore, physical activity increases the body's core temperature, which subsequently decreases post-exercise; this in turn can facilitate the onset of sleep, as the decrease in body temperature is a physiological indicator that it is time to sleep (54). Finally, physical activity can help regulate body weight; Indeed, obesity and related conditions, such as sleep apnea, can negatively impact sleep quality (55).

Finally, in the current study, physical activity and sleep hygiene knowledge explained 17.9% of the variability in sleep duration. This finding indicates that nearly one-fifth of the variability in the duration of sleep among students can be attributed to these two factors, which underscoring their importance in promoting adequate sleep. This result is noteworthy because it demonstrates the collective impact of physical activity and knowledge of sleep hygiene on the duration of sleep. Regular physical activity can be posited to contribute to longer sleep duration (26). On the other hand, an understanding of sleep hygiene enables students to implement practices that enhance the duration and quality of their sleep; these include maintaining regular sleep schedules, avoiding stimulants before bedtime, and creating an environment conducive to rest. Consequently, encouraging regular physical activity and providing education on healthy sleep practices represent an effective strategy to improve the health and well-being of students.

While this study provides important findings on the factors associated with sleep quality among nursing and medical students in Chiclayo, Peru, it is essential to consider how these results might vary across different populations and cultural contexts. For instance, students in non-health-related disciplines may encounter distinct stressors, academic workloads, and lifestyle factors that could influence the relationships observed between sleep hygiene knowledge, physical activity, and sleep quality (56). Additionally, cultural norms and socioeconomic conditions may play a significant role in shaping attitudes toward sleep hygiene and physical activity, potentially leading to different outcomes (57). Future studies should investigate these variables in diverse settings and among various student populations to better understand the broader applicability of these findings and to identify unique challenges and opportunities in promoting sleep quality.

### Public health and clinical implications

These findings have several implications from a public health and clinical perspective. In the field of public health, the findings underscore the necessity of developing and implementing educational programs that promote both physical activity and sleep hygiene knowledge among university students, particularly those who pursue demanding fields such as nursing and medicine. Furthermore, these findings indicate that university institutional policies should incorporate the integration of adequate breaks and physical activities into the academic schedule. This could include scheduling “active breaks” during the day, creating accessible spaces for physical activity on campus, and promoting an environment that supports healthy sleep habits. In this way, educational institutions can facilitate the enhancement of their students' general well-being, which is of significant impact for their academic performance and subsequent professional development.

From a clinical perspective, these findings highlight the importance of assessing and addressing physical activity and sleep habits in routine student health assessments. Health professionals should be trained to identify poor sleep patterns and insufficient levels of physical activity, providing appropriate advice and resources to improve these aspects. It is recommended that university clinics and student health services implement specific interventions, such as sleep hygiene workshops and exercise programs, to support students in adopting healthier habits.

### Limitations and future directions

Despite the positive and significant association found between sleep hygiene knowledge, physical activity, and sleep quality, it is important to consider several limitations when interpreting the results of the current study. First, the cross-sectional and observational nature of the study precludes the establishment of causal relationships, thereby limiting our ability to definitively state that sleep hygiene knowledge and physical activity improve sleep quality. Second, the use of self-reported online surveys can result in the introduction of bias in the collected data. It is possible that participants may have provided responses that were socially desirable or inaccurate, which could affect the validity of the findings. Furthermore, the veracity of responses can fluctuate depending on the comprehension of the inquiries by the respondents.

Another limitation is the variability in exercise behavior throughout the academic year. Physical activity levels can fluctuate considerably depending on the time of the semester. For example, they can increase before or after examinations, or at the beginning or end of the semester (51). This could have introduced a degree of inaccuracy into the assessment of exercise, potentially affecting the observed associations between physical activity and sleep quality. Furthermore, the study was conducted at a single Peruvian university situated in Chiclayo, Peru, and the data were collected through non-probability convenience sampling. These limitations preclude the generalizability of the results to other university populations, both within and outside of Peru.

Additionally, the study did not collect information regarding the academic level of participants, such as whether they were enrolled in clinical internships or theoretical courses. This distinction may be relevant, as the academic demands of clinical rotations or intensive coursework could influence sleep quality and physical activity levels differently. Future studies should consider including this variable to provide a more nuanced understanding of the observed associations. Finally, more than half of the participants were women. This may have an impact on the results, as sleep quality and physical activity may vary by gender. It would be beneficial for future studies to consider including a more gender-balanced sample to gain a more comprehensive understanding of these associations.

For future research, it is recommended to use longitudinal designs that can provide information on the causal relationships between sleep hygiene knowledge, physical activity, and sleep quality. Furthermore, it would be advantageous to utilize more precise data collection techniques, such as physical activity tracking devices and sleep monitoring, to minimize the impact of self-report biases. On the other hand, although this study focused on the relationships between sleep hygiene knowledge, physical activity, and sleep quality, other factors may also influence these associations. Variables such as academic stress levels, dietary habits, pre-sleep electronic device use, and social support were not assessed in this study but could provide additional insights into the observed patterns. Finaly, extending the research to multiple universities and regions would facilitate the generalization of the results and provide a more comprehensive understanding of these phenomena in different educational and cultural contexts.

## Conclusion

In conclusion, both sleep hygiene knowledge and physical activity are significantly associated with sleep quality in nursing and medical students. This study highlights the importance of knowledge of sleep hygiene and physical activity in the sleep quality of nursing and medical students. The implementation of educational strategies and the promotion of healthy habits can have a significant impact on the well-being and academic performance of future health professionals. These findings highlight the need for interventions designed to improve sleep quality during periods of crisis and in the daily routines of health students.

## Data Availability

The raw data supporting the conclusions of this article will be made available by the authors, without undue reservation.
